# Time for a Break: Admissions to an Urban Emergency Department after Working Out—A Retrospective Study from Switzerland

**DOI:** 10.1155/2015/610137

**Published:** 2015-02-04

**Authors:** Valentina A. Imstepf, Christian T. Braun, Meret E. Ricklin, Aristomenis K. Exadaktylos

**Affiliations:** Department of Emergency Medicine, University Hospital and University of Bern, Freiburgstrasse 16c, 3010 Bern, Switzerland

## Abstract

*Background*. The present retrospective study was intended to investigate whether working out and other low-speed sports can provoke cardiovascular, neurological, or traumatic damage. *Material and Methods*. Patient data from 2007 to 2013 was collected and saved at the university department of emergency medicine in an electronic patient record database. *Results*. Of the 138 patients included in this study, 83.3% (*n* = 115) were male and 16.7% female (*n* = 23). Most admissions were due to musculoskeletal accidents (*n* = 77; 55.8%), followed by neurological incidents (*n* = 23; 16.7%), cardiovascular incidents (*n* = 19; 13.8%), soft tissue injuries (*n* = 3; 2.2%), and others (*n* = 16; 11.6%). The mean age of the allover injured people was 36.7 years. The majority of the patients (*n* = 113; 81.9%) were treated as outpatients; 24 (17.4%) were inpatients. *Discussion*. In Switzerland, this is the first study that describes emergency department admissions after workout and examines trauma and neurological and cardiovascular incidents. As specific injuries, such as brain haemorrhages, STEMIs, and epileptic seizures, were relatively frequent, it was hypothesised that workout with its physiological changes may be an actual trigger for these injuries, at least for a specific population. * Conclusion*. Strenuous physical activity may trigger the risk of cardiovascular, neurological, or trauma events.

## 1. Introduction

Working out and other sports, such as cycling, jogging, or swimming, are becoming more and more popular, not only among the young but also among the older generation [1]. Regular exercise is meant to protect our bodies from early degeneration and lifestyle diseases. Whereas sports and workout used to be simply a means to the end of maintaining a muscular body, exercise is now regarded as a tool to smooth the way to a healthy body in old age [2, 3]. Not only can the young profit by working out, but also the elderly benefit from modifications in their antioxidant defence systems and in their muscle characteristics such as strength and endurance, making them less susceptible to acute injury and chronic inflammation [4]. Even though life-long exercise failed to prolong the lifespan of mice, Garcia-Valles et al. in 2013 showed that regular exercise “can improve age-associated frailty and improves the functional state” [5].

The Swiss Survey for Health (SGB) of the Federal Statistical Office for 2002, 2007, and 2012 shows that, in 1978, approximately 20% of the population exercised regularly, meaning more often than twice a week, whereas in 2007 as many as 56% of the population performed exercises or workout, including 51% several times a week and 30% only once per week [6]. According to the data of the SGB 2012, the percentage of active men (76%) is slightly higher than the percentage of active women (69%) [6], but the percentage of fitness centre members in Switzerland is almost the same for the two genders (14% female, 13.2% male). 13.6% of the Swiss population are members of a fitness or sports centre [1].

It is well known that gentle and regular physical activity promotes good health. It is less clear whether a strenuous workout can also be the trigger for cardiovascular or neurological incidents. Working out is very popular, but there is little published data on the types of injuries or incidents among the participants.

We studied a sample of 138 adults admitted to the emergency department after workout and describe their injuries, as well as neurological and cardiovascular incidents. We wanted to see whether there is a specific population at risk or a pattern of specific injuries or incidents after workout.

## 2. Material and Methods

Our university department of emergency medicine, the only Level I trauma centre in the Canton of Bern, serves about 1.8 million people and treats more than 35,000 cases per year, caring for patients older than 16 years. From 2007 to 2013, patient data was collected and saved at the university department of emergency medicine in an electronic patient record database (ECARE/Qualicare). The database was browsed for the key words “Training,” “Hantel,” and “Fitness” (“workout,” “barbell,” and “fitness centre”) and manually analysed for its accuracy. Search terms also included workout, indoor cycling, treadmill, and swimming. In the database, we identified 138 patients who were admitted to the emergency department after workout, either immediately or, at the latest, one day after workout. Since this medical database allows instantaneous recall of past diagnostic reports, consultations, X-rays, and other relevant medical documents, the authors were able to analyse retrospectively the identified patients. We included all patients presenting to our emergency department (ED) during the study period and after performing the following sporting activities: workout, indoor cycling, treadmill, or swimming. We excluded any contact sports (e.g., boxing or karate), as well as high-speed sports (e.g., skiing/snowboarding). The data were classified by gender, age, outcome (outpatient, inpatient, and death), type of sport, and cardiovascular, neurological, and musculoskeletal events, soft tissue injuries, and “others,” such as hyperventilation and rhabdomyolysis.

## 3. Results

**Figure 1:**
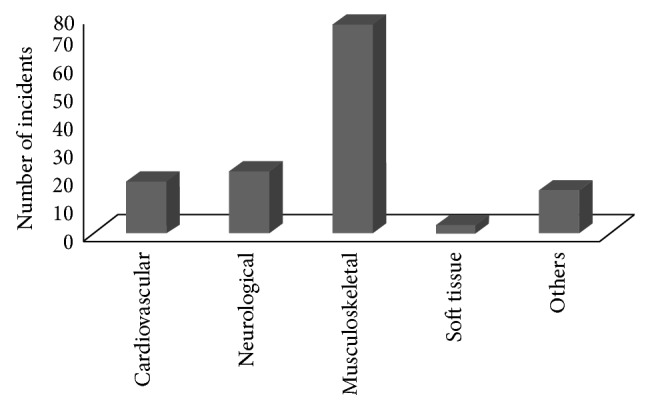
Number of incidents after workout subdivided into 5 groups according to the type of injury.

138 Patients were included in this study, including 115 men (83.3%) and 23 women (16.7%). We subdivided the 138 emergency department admissions into 5 subgroups: cardiovascular incidents (*n* = 19; 13.8%), neurological incidents (*n* = 23; 16.7%), musculoskeletal accidents (*n* = 77; 55.8%), soft tissue injuries (*n* = 3; 2.2%), and others (*n* = 16; 11.6%) (Figure 1).

**Figure 2:**
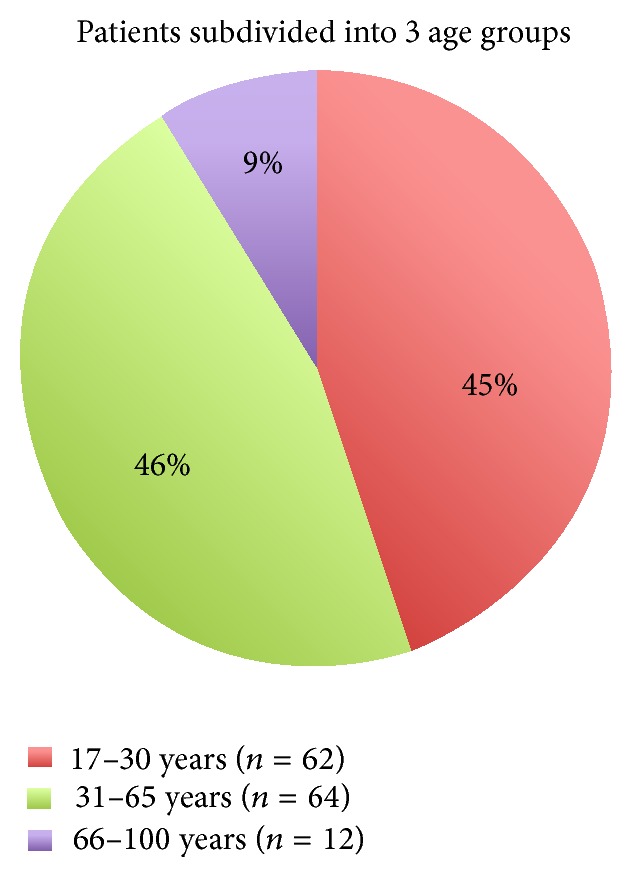
Patients with incidents after workout subdivided into 3 age groups.

The mean age of the injured people was 36.7 years.  Figure 2 shows the distribution of the patients with incidents after workout subdivided into 3 age groups: 45% (*n* = 62) of all patients belong to the age group of the young generation from 17 to 30 years; 46% (*n* = 64) belong to the generation aged between 31 and 65 years; and only 9% (*n* = 12) are older than 66 years.

The mean age of the patients was different for each category: for cardiovascular incidents, the mean age is 47.2 years, for neurological incidents 44.2 years, for musculoskeletal trauma 33.2 years, for soft tissue injuries 20.0 years, and for the “others” 33.1 years.

The main causes for emergency department admissions were musculoskeletal accidents (*n* = 77), followed by neurological events (*n* = 23) and cardiovascular events (*n* = 19). Soft tissue injuries (*n* = 3) and others causes such as infection, hyperventilation, and rhabdomyolysis (*n* = 16) were less significant.

**Figure 3:**
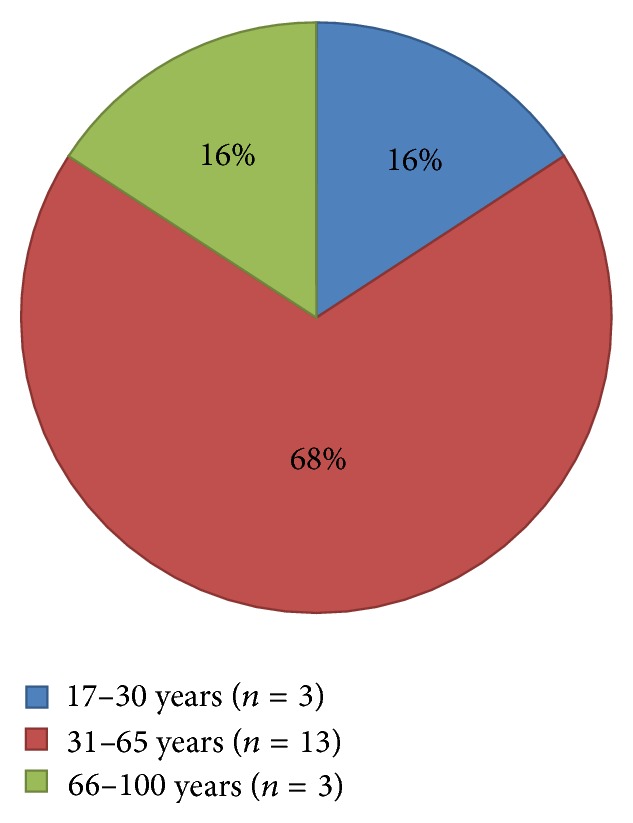
Cardiovascular events by age groups: the majority are male and 31–65 years old.

Division of the subcategories by gender and age groups (17–30 years; 31–65 years; 66–100 years) showed that, for cardiovascular events, males are predominant (males 89.5%; *n* = 17, females 10.5%; *n* = 2) and that most patients were between 31 and 65 years of age (68.4%; *n* = 13) (Figure 3). The leading causes in the group of cardiovascular events were cardiac arrhythmias (*n* = 7), followed by syncope and presyncope (*n* = 4), STEMIs (*n* = 3), and hypertensive crisis (*n* = 3); two of the latter patients had already been treated with antihypertensive medication. Carotid dissection, aortic dissection, and symptomatic varicosis of the limb were exceptional (Table 1). As predisposing factors in the age groups >30 years, seven out of sixteen patients suffered from hypertension, four patients were described with nicotine abuse, four suffered from dyslipidemia, and two suffered from hypercholesteremia. Three patients of the cardiovascular group were described with vessel coronary disease: two of them had a therapy with stent-implantation before and one had received a coronary artery bypass graft surgery before. 5 patients of the cardiovascular group with age > 30 years were treated before with inhibitor of platelet aggregation and 7 of them had an antihypertensive medication (Supplement 2 available online at http://dx.doi.org/10.1155/2015/610137); the other medication in the cardiovascular group was heterogenous (Supplement 2).

Males were also predominant in patients suffering from neurological events (males 82.6%; *n* = 19, females 17.4%; *n* = 4) and most of the patients were aged between 31 and 65 years (43.5%; *n* = 10), followed by 17–30 years (34.5%; *n* = 8) and 66–100 years (21.7%; *n* = 5). Most neurological events after workout were epileptic seizures (*n* = 5) (including two patients with prior epileptic episodes) and cerebral haemorrhage (*n* = 5), followed by ischaemic stroke (*n* = 3), transient global amnesia (*n* = 3), and other neurological events (*n* = 7), such as dizziness, migraine, craniocerebral injury, and unclear neurological symptoms (Table 2). Of the three patients who suffered an ischaemic stroke, two suffered from hypertension; one patient exhibited dyslipidemia and had a positive family history of IS, as well as a prior event of TIA and a CVI. None of these patients smoked.

Males were also predominant in the group of musculoskeletal accidents (males 83.1%; *n* = 64, females 16.9%; *n* = 13) but were clearly younger than the other two groups, most being between 17 and 30 years old (52.0%; *n* = 40) (Figure 5). In this group, musculoskeletal pain syndrome (*n* = 43) is the leading cause for emergency department admissions. This was followed by distortion, dislocation, torn ligament, tendinitis (*n* = 13), fractures (*n* = 6), contusion and open wound (*n* = 3), and others (*n* = 12) (Table 3).

Most of the incidents of all types were due to workout (*n* = 120; 87%), followed by treadmill (*n* = 8; 5.8%), indoor cycling (*n* = 7; 5.1%), and swimming (*n* = 3; 2.2%).

The largest class of events after indoor cycling were neurological (*n* = 4; 17.4%) and cardiovascular (*n* = 3; 15.8%) incidents. Workout was the main source of emergency department admissions in all 5 subgroups. Treadmill just figured in the subgroup of musculoskeletal accidents (10.4%). Events related to swimming were found in the cardiovascular (10.5%) and the “other” (6.3%) subgroups.

The largest section of patients (*n* = 113; 81.9%) was treated as outpatients; 24 patients were treated as inpatients (17.4%); and there was one death (0.7%) from a pericardial tamponade after a type A aortic dissection.

## 4. Discussion

### 4.1. Cardiovascular Incidents

It is known that regular physical activity in both men and women can decrease rates of coronary heart disease and cardiovascular disease in general [7, 8]. However, there are qualifications to this conclusion. Willich et al. showed in 1993 not only that there is an augmented risk of acute myocardial infarction during or shortly after vigorous physical exercise, but also that the individual baseline level of physical fitness is a factor determining the risk of a cardiovascular event during exercise [9]. After moderate or exhausting exercise, the main period of risk for developing symptoms of acute myocardial infarction (AMI) is 2 hours [10]. Moreover the same study showed that there is a correlation between the intensity of the physical activity, the age of the subject, and the risk of developing symptoms of acute MI [10]. The risk of cardiovascular “side effects” is pronounced in strenuous exercise, whereas moderate exercise is advantageous for cardiovascular morbidity and mortality [11]. These findings could explain the increase in STEMIs and arrhythmias immediately after or during exercise; this applied to people aged 31 to 65 years but less to younger people aged 17 to 30 (Figure 3, Table 1). Moreover, there were fewer events in the age group from 66 to 100 years, probably because there were fewer people in this age group working out in a fitness centre. Even though the intensity of the exercise was not classified, clear criteria would be needed, as each subject has different baseline levels of physical fitness which can prevent these cardiovascular “side effects” by overcharging the untrained body.

Furthermore there seems to be an increased risk of acute MI in the 3-hour period following waking up [9]. This increased risk has its origin not only in increased morning blood pressure or increased heart rate after rising [12], but also due to a procoagulative state with increased platelet aggregability, accompanied by decreased fibrinolytic activity [13]. This diminished fibrinolytic activity leads to an increased risk of blood clotting. It is subject to circadian rhythms and is also present in people who do not exercise regularly [14]. People who do not exercise regularly or do not exercise at all have got a 26-fold increase in the risk of MI onset on performing strenuous exercise and have got a 3.5-fold increase in the risk of MI onset on performing moderate exercise [10]. These different findings might lead to the recommendation that people who do not exercise regularly should not perform vigorous exercise or work out in the 3-hour period after waking up.

### 4.2. Neurological Incidents

Whereas there seems to be clear evidence for an increased risk in onset of AMI after moderate to strenuous exercise, mostly for untrained subjects, the risk of a cerebrovascular insult (CVI) linked to heavy exercise is controversial. Subarachnoid haemorrhage (SAH) and ischaemic stroke (IS) must be distinguished in this context.

### 4.3. Subarachnoid Haemorrhage

Anderson et al. studied a group of patients with the first event of SAH, of which 76% presented an aneurysm as origin of the bleeding. By analysing the activities at the very moment of the SAH or the activities preceding the haemorrhage, they could show that, in people who engaged in moderate to heavy physical activity, there is a threefold risk for SAH in a time window of the following 2 hours, when compared to periods of low physical activity [15]. Fann et al. showed a “strong association” between heavy physical exertion and spontaneous SAH [16]. While it is known that hypertension is one of the major risk factors of aneurysm formation, it does not seem to be the leading cause of aneurysmal rupture [17], so there must be some other accompanying factors which lead to rupture and not just the acute increase in blood pressure during heavy exercise.

### 4.4. Ischaemic Stroke

As with the increased risk of MI in the morning hours [12, 13], there is a morning peak for IS [18]. In addition to this circadian risk, acute events such as vigorous physical exercise could act as trigger by simulating the same acute physiological changes as found in the morning peak, thereby increasing the risk of rupture of vulnerable plaque [19, 20].

### 4.5. Epileptic Seizure

**Figure 4:**
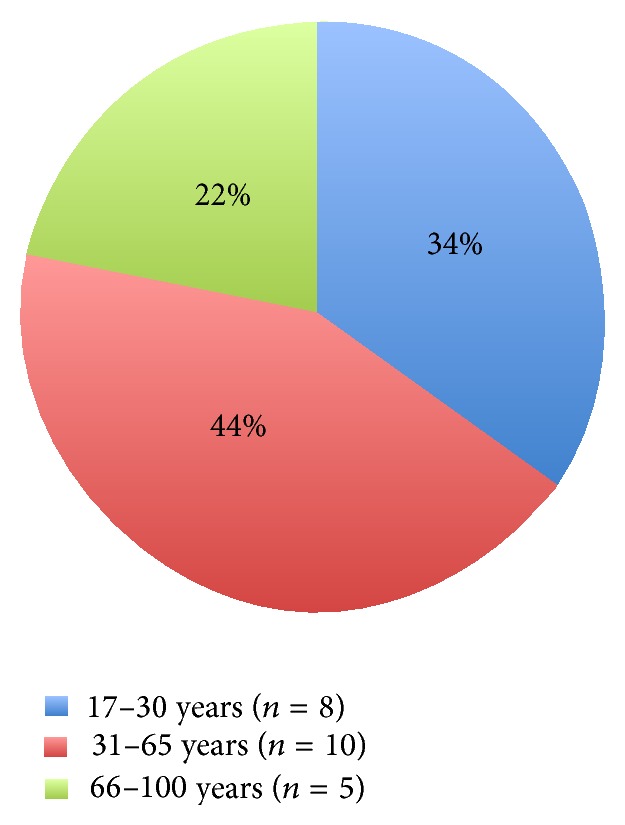
Neurological events by age groups: the majority are male and 31–65 years old.

As an epileptic seizure seems to be one of the leading neurological problems resulting in emergency department admission after workout (see Figure 4; Table 2), it was considered whether the workout was a trigger for epileptic seizures. In current scientific literature, exercise for patients with epilepsy is still a controversial issue: some studies claim that exercise is not a typical inducer for epileptic seizures [21–23] but is rather protective; even though the exact mechanism is not known exactly, exercise for people suffering from epilepsy seems to exert “favourable social, psychological, and physiological effects” [23]. There seems to be less evidence for exercise-induced seizures, and if so, these are more closely associated with strenuous exercise [22]. Therefore people with epileptic seizures should not be advised to avoid physical exercise [24], even though it is important to be aware of their individual medical history before giving this advice. It would be necessary to conduct further studies with a control group to establish whether in our case the accumulation of epileptic seizures was a coincidence or whether other environmental factors could have played a role.

### 4.6. Trauma

**Figure 5:**
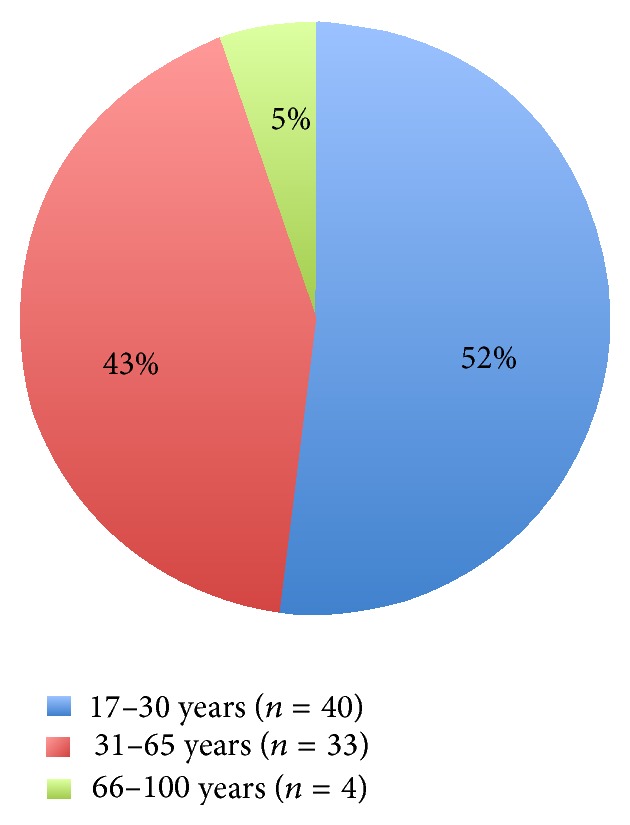
Musculoskeletal events by age groups: the majority are male and 17–30 years old.

In our study, the subgroup of trauma was the leading cause (*n* = 77; 55.8%) of emergency department admissions after workout (Figure 1). Moreover, trauma was associated with the youngest, with 52% in the age group from 17 to 30 years and 43% in the age group from 31 to 65 years (Figure 5; Table 3). As in our study, the German national health survey shows that young males are at the greatest risk of sports injuries, whereas females are at less risk [25]. Loose ligaments could play an important role in sports injuries of young adults: this was shown to be one reason for an increased risk of musculoskeletal injuries (mainly in young males), where the lower limbs were more often affected than the upper limbs [26]. Old or middle-aged people are often nervous about sports injuries and therefore reduced their activity [27], but their anxieties may be unjustified: it has been shown that older adults do not have greater risks of sports injuries than younger adults [28]. Our data confirm these findings, as trauma in the 66–100-year-old group makes up only 5% of the total traumatic injuries (even though in this context it has to be considered that the age group from 66 to 100 years less often frequents a fitness centre for workout) (Figure 5).

In our study, most of the emergency department admissions after trauma were due to musculoskeletal pain syndromes (*n* = 43), followed by distortion, dislocation, torn ligament, tendinitis (*n* = 13), fractures (fracture of fingers: *n* = 5; fracture of nose: *n* = 1), contusions and open wounds (*n* = 3), and others (*n* = 12) (Table 3). Schneider et al. found that distortion, dislocation, and torn ligaments were the leading cause of sports injuries (60%), with fractures in the second place (18%) [29].

Furthermore it would be interesting to know about the frequency of workouts, as some studies show an association between sports injuries and training sessions per week [30, 31].

## 5. Limitations

Our findings have to be considered with some caution, as the study was conducted retrospectively. As the information in our medical history database is presented in a narrative comment, there is no guarantee that the number of patients was fully reported. Furthermore, our study was limited to adults (≥16 years old), as children are treated at a separate emergency department in our hospital. We think, however, that the retrospective time window from 2007 to 2013 balances the seasonal variations for injury likelihoods and environmental changes.

Furthermore our study population is not balanced with respect to age or gender distribution, so that there might be a bias regarding the younger and male patients. Generally it is difficult to compare groups of different ages, as they differ greatly in terms of underlying diseases or general body condition. In a follow-up study, it would be interesting to describe the exact time (morning versus evening) of the exercise, the general condition of the participants (underlying diseases, risk factors or prior medical conditions), the intensity level of the exercise, the regularity and duration of physical activity or practice, the type of exercise, and documentation of any warming up period.

## 6. Conclusions

### 6.1. Cardiovascular Incidents

Workout and other low-speed sports seem to be protective for human health, when performed regularly and cautiously. Strenuous physical activity however may exacerbate the risks of cardiac death and myocardial infarction, mainly for the untrained people and people at some cardiovascular risk (due to degeneration and/or lifestyle) or people with occult heart disease [10, 32, 33].

### 6.2. Neurological Incidents

For the neurological events, the younger (17–30 years and 31–65 years) groups are more at risk than the oldest group (66–100 years). One reason is that the older generation in general works out less often than the younger generation [27]. Even though exercise-induced epileptic seizures are rather rare [23], in our analysis it was the leading cause (together with cerebral haemorrhages) of admissions to the neurological emergency department.

### 6.3. Trauma

Our study shows that the younger male generation (with 52% in the group of 17–30 years) has a pronounced risk of traumatic injury during workout. One reason could be that ligaments and bones are less stable in adolescents, who are then more susceptible to trauma [26]. Furthermore there is evidence that warming up before physical exercise may reduce the risk of trauma [34, 35], whereas the importance of stretching before and/or after the exercise remains debatable [36–38].

## Supplementary Material

Supplement 1: Detailed information about all patients regarding sex, age, region of injury, direct trauma and outcome classified as cardiovascular or neurological incident, musculoskeletal injury, soft tissue injury and others.
Supplement 2: Detailed information about risk factors, prior events and medical treatment of patients in the cardiovascualr group aged > 30 years and the neurological patients presenting with IS or epileptic seizures.

## Figures and Tables

**Table 1:** Cardiovascular events divided into subgroups.

Cardiovascular event	Number
Cardiac arrhythmia	6
Syncope	4
STEMI	3
Hypertensive crisis	3
Dissection events	3

**Table 2:** Neurological events divided into subgroups.

Neurological event	Number
Epileptic seizure	5
Cerebral haemorrhage	5
Ischaemic stroke	3
Transient global amnesia	3
Other neurological events	7

**Table 3:** Musculoskeletal events divided into subgroups.

Musculoskeletal event	Number
Musculoskeletal pain syndrome	43
Distortion, dislocation, torn ligament, tendinitis	13
Fracture	6
Contusion, open wound	3
Other injuries	12

## Abbreviations

ED: Emergency department
SAH: Subarachnoid haemorrhage
SAB: Subarachnoid bleeding
MI: Myocardial infarction
IS: Ischaemic stroke
SGB: Swiss Survey for Health
CVI: Cerebrovascular insult
STEMI: ST-elevations myocardial infarction
Fx: Fracture.

## References

[1] Lamprecht M., Fischer A., Stamm H. Sport Schweiz 2008: Das Sportverhalten der Schweizer Bevölkerung 2008. http://www.baspo.admin.ch/internet/baspo/de/home/dokumentation.parsys.0001101.downloadList.17485.DownloadFile.tmp/basposportschweizde.pdf.

[2] Warburton D. E. R., Nicol C. W., Bredin S. S. D. (2006). Health benefits of physical activity: the evidence. *Canadian Medical Association Journal*.

[3] Kerr J., Rosenberg D., Frank L. (2012). The role of the built environment in healthy aging community design, physical activity, and health among older adults. *Journal of Planning Literature*.

[4] Ji L. L. (2001). Exercise at old age: does it increase or alleviate oxidative stress?. *Annals of the New York Academy of Sciences*.

[5] Garcia-Valles R., Gomez-Cabrera M. C., Rodriguez-Mañas L. (2013). Life-long spontaneous exercise does not prolong lifespan but improves health span in mice. *Longevity & Healthspan*.

[6] Lamprecht M., Stamm H., Fischer A., Gebert A., Wiegand D. Observatorium Sport und Bewegung Schweiz, Laufend aktualisierte Indikatoren. http://www.sportobs.ch/fileadmin/sportobs-dateien/Indikatoren_PDF/SPORTOBS_Updated.pdf.

[7] Shiroma E. J., Lee I.-M. (2010). Physical activity and cardiovascular health: lessons learned from epidemiological studies across age, Gender, and race/ethnicity. *Circulation*.

[8] Ignarro L. J., Balestrieri M. L., Napoli C. (2007). Nutrition, physical activity, and cardiovascular disease: an update. *Cardiovascular Research*.

[9] Willich S. N., Lewis M., Lowel H., Arntz H.-R., Schubert F., Schroder R. (1993). Physical exertion as a trigger of acute myocardial infarction. *New England Journal of Medicine*.

[10] Von Klot S., Mittleman M. A., Dockery D. W. (2008). Intensity of physical exertion and triggering of myocardial infarction: a case-crossover study. *European Heart Journal*.

[11] Dangardt F. J., McKenna W. J., Lüscher T. F., Deanfield J. E. (2013). Exercise: friend or foe?. *Nature Reviews Cardiology*.

[12] Muller J. E., Tofler G. H., Stone P. H. (1989). Circadian variation and triggers of onset of acute cardiovascular disease. *Circulation*.

[13] Andreotti F., Davies G. J., Hackett D. R. (1988). Major circadian fluctuations in fibrinolytic factors and possible relevance to time of onset of myocardial infarction, sudden cardiac death and stroke. *The American Journal of Cardiology*.

[14] Speiser W., Langer W., Pschaick A. (1988). Increased blood fibrinolytic activity after physical exercise: comparative study in individuals with different sporting activities and in patients after myocardial infarction taking part in a rehabilitation sports program. *Thrombosis Research*.

[15] Anderson C., Mhurchu C. N., Scott D., Bennett D., Jamrozik K., Hankey G. (2003). Triggers of subarachnoid hemorrhage: role of physical exertion, smoking, and alcohol in the Australasian Cooperative Research on Subarachnoid Hemorrhage Study (ACROSS). *Stroke*.

[16] Fann J. R., Kukull W. A., Katon W. J., Longstreth W. T. (2000). Physical activity and subarachnoid haemorrhage: a population based case-control study. *Journal of Neurology Neurosurgery and Psychiatry*.

[17] Inagawa T. (2010). Risk factors for the formation and rupture of intracranial saccular aneurysms in Shimane, Japan. *World Neurosurgery*.

[18] Stergiou G. S., Vemmos K. N., Pliarchopoulou K. M., Synetos A. G., Roussias L. G., Mountokalakis T. D. (2002). Parallel morning and evening surge in stroke onset, blood pressure, and physical activity. *Stroke*.

[19] Tofler G. H., Muller J. E. (2006). Triggering of acute cardiovascular disease and potential preventive strategies. *Circulation*.

[20] Muller J. E., Abela G. S., Nesto R. W., Tofler G. H. (1994). Triggers, acute risk factors and vulnerable plaques: the lexicon of a new frontier. *Journal of the American College of Cardiology*.

[21] Arida R. M., Scorza F. A., dos Santos N. F., Peres C. A., Cavalheiro E. A. (1999). Effect of physical exercise on seizure occurrence in a model of temporal lobe epilepsy in rats. *Epilepsy Research*.

[22] Nakken K. O. (1999). Clinical research physical exercise in outpatients with epilepsy. *Epilepsia*.

[23] Nakken K. O., Bjørholt P. G., Johannessen S. I., Loyning T., Lind E. (1990). Effect of Physical training on aerobic capacity, seizure occurrence, and serum level of antiepileptic drugs in adults with epilepsy. *Epilepsia*.

[24] Arida R. M., Cavalheiro E. A., da Silva A. C., Scorza F. A. (2008). Physical activity and epilepsy: proven and predicted benefits. *Sports Medicine*.

[25] Bellach B. M., Knopf H., Thefeld W. (1998). The German health survey. 1997/98. *Gesundheitswesen*.

[26] Bin Abd Razak H. R., Bin Ali N., Howe T. S. (2014). Generalized ligamentous laxity may be a predisposing factor for musculoskeletal injuries. *Journal of Science and Medicine in Sport*.

[27] Crombie I. K., Irvine L., Williams B. (2004). Why older people do not participate in leisure time physical activity: a survey of activity levels, beliefs and deterrents. *Age and Ageing*.

[28] Little R. M. D., Paterson D. H., Humphreys D. A., Stathokostas L. (2013). A 12-month incidence of exercise-related injuries in previously sedentary community-dwelling older adults following an exercise intervention. *BMJ Open*.

[29] Schneider S., Seither B., Tönges S., Schmitt H. (2006). Sports injuries: population based representative data on incidence, diagnosis, sequelae, and high risk groups. *British Journal of Sports Medicine*.

[30] Inouye J., Nichols A., Maskarinec G., Tseng C.-W. (2013). A survey of musculoskeletal injuries associated with Zumba. *Hawai'i Journal of Medicine & Public Health*.

[31] Campoy F. A. S., Coelho L. R., Bastos F. N. (2011). Investigation of risk factors and characteristics of dance injuries. *Clinical Journal of Sport Medicine*.

[32] Mittleman M. A., Maclure M., Tofler G. H., Sherwood J. B., Goldberg R. J., Muller J. E. (1993). Triggering of acute myocardial infarction by heavy physical exertion—protection against triggering by regular exertion. *New England Journal of Medicine*.

[33] Thompson P. D. (2003). Exercise and physical activity in the prevention and treatment of atherosclerotic cardiovascular disease. *Arteriosclerosis, Thrombosis, and Vascular Biology*.

[34] Herman K., Barton C., Malliaras P., Morrissey D. (2012). The effectiveness of neuromuscular warm-up strategies, that require no additional equipment, for preventing lower limb injuries during sports participation: a systematic review. *BMC Medicine*.

[35] Fradkin A. J., Gabbe B. J., Cameron P. A. (2006). Does warming up prevent injury in sport?. The evidence from randomised controlled trials?. *Journal of Science and Medicine in Sport*.

[36] Hart L. (2005). Effect of stretching on sport injury risk: a review. *Clinical Journal of Sport Medicine*.

[37] Thacker S. B., Gilchrist J., Stroup D. F., Kimsey C. D. (2004). The impact of stretching on sports injury risk: a systematic review of the literature. *Medicine and Science in Sports and Exercise*.

[38] Shellock F. G., Prentice W. E. (1985). Warming-up and stretching for improved physical performance and prevention of sports-related injuries. *Sports Medicine*.

