# Supplementary material for: Time for a Break: Admissions to an Urban Emergency Department after Working Out—A Retrospective Study from Switzerland
Source: Biomed Res Int. 2015 Feb 4;2015:610137. doi: 10.1155/2015/610137 (PMC4334856; doi:10.1155/2015/610137)
Supplement: Supplementary file 1 — Supplement 1: Detailed information about all patients regarding sex, age, region of injury, direct trauma and outcome classified as cardiovascular or neurological incident, musculoskeletal injury, soft tissue injury and others. Supplement 2: Detailed information about risk factors, prior events and medical treatment of patients in the cardiovascualr group aged > 30 years and the neurological patients presenting with IS or epileptic seizures. [file 610137.f1.zip › SUPPLEMENT 1.pdf]

SUPPLEMENT 1

Patients (n)	Sex	Age	Cause of accident	Region of injury	Direct Trauma	Outcome
**Cardiovascular**						
**1**	m	62	Workout	Atrial fibrillation	-	inpatient
**2**	m	64	Indoor cycling	STEMI	-	inpatient
**3**	m	38	Workout	Syncope with arterial<br><br>hypertension	-	outpatient
**4**	m	43	Workout	Uncontrolled hypertension with dyspnoea and leg oedema	-	outpatient
**5**	m	68	Workout	Syncope	-	outpatient
**6**	m	39	Swimming	Arrhythmia	-	inpatient
**7**	m	52	Workout	STEMI	-	inpatient
**8**	m	46	Indoor cycling	Several pre-syncopes	-	outpatient
**9**	m	17	Workout	Syncope with palpitations	-	outpatient
**10**	m	69	Swimming	TdP-Tachycardia		inpatient
**11**	m	37	Workout	Art. hypertension, palpitation and syncope	-	outpatient
**12**	m	40	Workout	Tachycardia and dyspnoea	-	outpatient
**13**	m	57	Workout	Syncope	-	outpatient
**14**	m	55	Workout	Carotid dissection	-	inpatient
**15**	m	68	Indoor cycling	STEMI	-	inpatient
**16**	f	29	Workout	Symptomatic varicosis	-	outpatient
**17**	f	53	Workout	Narrow complex tachycardia	-	outpatient

**18**	m	28	Workout	Death (type A dissection)	-	death
**19**	m	32	Workout	Atrial tachycardia	-	inpatient
**Neurological**						
**20**	m	46	Workout	IS	-	inpatient
**21**	m	68	Workout	IS	-	inpatient
**22**	m	79	Indoor cycling	Subarachnoid haemorrhage	-	inpatient
**23**	m	66	Workout	IS	-	inpatient
**24**	f	84	Workout	Cerebral haemorrhage	-	inpatient
**25**	m	39	Workout	Cerebral haemorrhage	-	Inpatient
**26**	m	67	Workout	Migraine	-	inpatient
**27**	f	50	Workout	Transient global amnesia	-	outpatient
**28**	m	40	Workout	Craniocerebral injury	Fall from pull-up-bar	outpatient
**29**	m	59	Workout	Transient global amnesia	-	outpatient
**30**	m	18	Indoor cycling	Epileptic seizure	-	inpatient
**31**	m	45	Workout	Epileptic seizure	-	outpatient
**32**	m	22	Workout	Migraine	-	outpatient
**33**	m	27	Workout	Subarachnoid haemorrhage	-	inpatient
**34**	m	47	Indoor cycling	Subarachnoid haemorrhage	-	inpatient
**35**	m	64	Workout	Epileptic seizure	-	inpatient
**36**	m	22	Workout	Epileptic seizure	-	inpatient
**37**	f	23	Workout	Dizziness	-	outpatient
**38**	m	18	Workout	Dizziness	-	outpatient
**39**	m	35	Workout	Unclear neurological picture	-	inpatient
**40**	m	19	Workout	Unclear neurological picture	-	inpatient

**41**	f	59	Home trainer	Transient global amnesia	-	outpatient
**42**	m	19	Workout	Epileptic seizure	-	inpatient
**Musculoskeletal**						
**43**	m	18	Workout	Chest pain	yes	outpatient
**44**	m	33	Workout	Metacarpal V Fx	yes	outpatient
**45**	m	39	Workout	Knee torsion	yes	outpatient
**46**	m	23	Treadmill	Musculotendinous overload ankle	yes	outpatient
**47**	m	20	Workout	Fx Phalanx	yes	outpatient
**48**	m	34	Workout	Tendovaginitis	yes	outpatient
**49**	f	34	Workout	Tendinitis calcarea	yes	outpatient
**50**	f	31	Workout	Ruptured muscle fibre	yes	outpatient
**51**	m	41	Workout	Insertion tendinitis M. Deltoideus	yes	outpatient
**52**	f	38	Treadmill	Runner’s knee	yes	outpatient
**53**	m	34	Workout	Soft tissue swelling hip	yes	outpatient
**54**	m	18	Workout	Nasal bone Fx	Hit nose	outpatient
**55**	m	20	Work-out	Amputation finger tip	Barbell fell on finger	outpatient
**56**	m	24	Workout	Chest pain	yes	outpatient
**57**	m	27	Workout	Contusion trochanter major	yes	outpatient
**58**	f	76	Workout	Hardening M. Trapezius	yes	outpatient
**59**	f	76	Workout	Hardening M. Trapezius	yes	outpatient
**60**	m	25	Workout	Lumbago	yes	outpatient
**61**	m	38	Treadmill	Lesion of lateral meniscus		outpatient
**62**	m	18	Workout	Contusion upper arm	yes	outpatient
**63**	m	29	Workout	Hardening M. gluteus	yes	outpatient

**64**	m	25	Workout	Distortion ankle	yes	outpatient
**65**	m	17	Workout	Metacarpal I Fx	Barbell fell on hand	outpatient
**66**	m	19	Workout	Hardening muscles neck	yes	outpatient
**67**	m	33	Workout	Tendinitis M. supraspinatus	yes	outpatient
**68**	m	79	Workout	Pain vertebral bodies	yes	outpatient
**69**	m	19	Workout	Headache and pain vertebral bodies	yes	outpatient
**70**	m	44	Treadmill	Herniated disc	yes	outpatient
**71**	m	26	Workout	Headache	yes	outpatient
**72**	m	25	Workout	Muscular stiffness thorax	yes	outpatient
**73**	m	30	Workout	Subacromial impingement	yes	outpatient
**74**	m	34	Workout	Lumboischialgia	yes	outpatient
**75**	m	23	Workout	Hardening M. trapezius	yes	outpatient
**76**	m	22	Workout	Tendinitis M. extensor pollicis longus	yes	outpatient
**77**	m	26	Workout	Paraesthesia finger tips I-V	yes	outpatient
**78**	f	44	Workout	Activated osteochondrosis	yes	outpatient
**79**	m	48	Workout	Strain trauma adductor muscles	yes	outpatient
**80**	m	24	Treadmill	Overload tendinitis condylus lat. knee	yes	outpatient
**81**	f	23	Workout	Lumbosacral pain syndrome	yes	outpatient
**82**	m	54	Workout	Insertion tendinitis M. serratus	yes	outpatient
**83**	m	45	Workout	Pseudoradicular syndrome	yes	outpatient
**84**	m	38	Workout	Mechanical carpal tunnel syndrome	yes	outpatient

**85**	m	36	Workout	Pain lower limb	yes	outpatient
**86**	f	24	Workout	Lumbalgia with pseudoradicular pain	yes	outpatient
**87**	m	84	Treadmill	Bursitis trochanterica	yes	outpatient
**88**	m	34	Treadmill	Tendovaginitis foot	yes	outpatient
**89**	m	29	Workout	Acute lumbalgia	yes	outpatient
**90**	f	27	Workout	Musculoskeletal pain sternum	yes	outpatient
**91**	m	21	Workout	Subluxation shoulder	Lifting weights	outpatient
**92**	m	56	Workout	Luxation shoulder	yes	outpatient
**93**	m	44	Workout	Musculoskeletal pain	yes	outpatient
**94**	m	18	Workout	Tension headache	yes	outpatient
**95**	m	27	Workout	Lumbago	Lifting weights	outpatient
**96**	m	28	Workout	Luxation shoulder	Barbell slipped away	outpatient
**97**	m	25	Workout	Exercise induced headache	yes	outpatient
**98**	f	37	Workout	Muscular neck pain	yes	outpatient
**99**	f	25	Workout	Muscular neck pain	yes	outpatient
**100**	f	42	Workout	Muscular chest pain		outpatient
**101**	m	18	Workout	Rupture of muscle fibre quadriceps	yes	outpatient
**102**	m	55	Workout	Swollen ankle (overuse)	yes	outpatient
**103**	m	42	Workout	Musculoskeletal chest pain	yes	outpatient
**104**	m	35	Workout	Pain coccyx (overload)	yes	outpatient
**105**	m	17	Workout	Overuse biceps tendon	yes	outpatient
**106**	m	37	Workout	Tendinitis M. quadriceps	yes	outpatient

**107**	m	63	Workout	Musculoskeletal chest pain	yes	outpatient
**108**	m	27	Workout	Cervical pain syndrome	yes	outpatient
**109**	m	33	Workout	Finger tip Fx	yes	outpatient
**110**	m	18	Workout	Inguinal hernia	yes	outpatient
**111**	m	46	Workout	Musculoskeletal chest pain	yes	outpatient
**112**	m	27	Workout	Musculoskeletal pain M. pectoralis & M. SCM	yes	outpatient
**113**	m	24	Workout	Musculoskeletal pain hip and shoulder	yes	outpatient
**114**	m	31	Treadmill	Tendinitis Achilles tendon	yes	outpatient
**115**	m	17	Workout	Contusion toe I + II	Barbell fell on toes	outpatient
**116**	m	23	Workout	Shoulder luxation	Barbell fell down	outpatient
**117**	m	29	Workout	Cervical myogelosis	yes	outpatient
**118**	m	31	Workout	Cervical syndrome	yes	outpatient
**119**	m	31	Workout	Trauma of thumb	yes	outpatient
**Soft Tissue Injuries**						
**120**	m	19	Workout	Laceration cheek	Barbell fell on cheek	outpatient
**121**	m	24	Workout	Laceration eye	Barbell fell on eye	outpatient
**122**	m	17	Workout	Laceration tibia	Hit leg on a iron rod	outpatient
**Others (Infections, Rhabdomyolysis...)**						
**123**	m	39	Workout	Pneumonia	-	outpatient
**124**	f	39	Workout	Urticaria	-	outpatient
**125**	m	45	Workout	Urolithiasis	-	outpatient
**126**	m	44	Swimming	Nausea, vomiting	overexertion	outpatient
**127**	f	22	Workout	Tinnitus	-	outpatient

**128**	f	30	Workout	Exercise-induced Asthma	yes	outpatient
**129**	m	20	Workout	Vomiting blood	-	outpatient
**130**	m	28	Workout	Red-brown urine and high CK	yes	outpatient
**131**	m	37	Workout	Hyperventilation after taking anabolic steroids		outpatient
**132**	m	29	Workout	Unclear sore throat	-	outpatient
**133**	m	32	Workout	Rhabdomyolysis	-	inpatient
**134**	f	40	Workout	Acute hyperventilation	-	outpatient
**135**	m	18	Workout	Unclear headache	-	outpatient
**136**	m	27	Workout	Acute rhabdomyolysis	-	outpatient
**137**	m	51	Workout	Unclear thoracic paraesthesia	-	outpatient
**138**	m	29	Workout	Somatisation trouble and hyperventilation	-	outpatient
