# Supplementary material for: Time for a Break: Admissions to an Urban Emergency Department after Working Out—A Retrospective Study from Switzerland
Source: Biomed Res Int. 2015 Feb 4;2015:610137. doi: 10.1155/2015/610137 (PMC4334856; doi:10.1155/2015/610137)
Supplement: Supplementary file 1 — Supplement 1: Detailed information about all patients regarding sex, age, region of injury, direct trauma and outcome classified as cardiovascular or neurological incident, musculoskeletal injury, soft tissue injury and others. Supplement 2: Detailed information about risk factors, prior events and medical treatment of patients in the cardiovascualr group aged > 30 years and the neurological patients presenting with IS or epileptic seizures. [file 610137.f1.zip › SUPPLEMENT 2.pdf]

Cardiovascular events (Patient groups > 30 years)

Patient number	Problem	Smoking	Hypertension	Dyslipidemia	Hyper-cholesterinemia	Underlying disease or prior events	Family history	Medication
**1**	Atrial Fibrillation	x (30py)	x			Stent RCA, RCX, IMA		Aspirin cardio 100mg ; Nebilet 5mg ; Co-Aprovel 150mg
**2**	STEMI		x					
**3**	Syncope with arterial hypertension		**x**			Hyper-tension		Lisopril plus 20mg; Amlodipin 10mg
**4**	Uncontrolled hypertension with dyspnoea and leg oedema	x			x	None		Brufen 600mg
**5**	Syncope	2 pipes/week	x	x	x	diastolic dysfunction; left atrial and ventricuar dilatation with normal LVEF		Xenalon 50; Aspirin 100mg; Tamsulosim 0.4, Sortis 40mg; Votum 20; Dilzem 90mg
**6**	Arrhytmia of unknown origin		Yes, but normotone with ACE-inhibitors					Coversum 8 mg; Neurontin 800 mg
**7**	STEMI	x		x				
**8**	Several Pre-syncopes	none						
**10**	TdP-Tachykardia		x	x				Metozerok 50 mg, Atacand; cholesterol-lowering agent

**Patient number**	**Problem**	**Smoking**	**Hypertension**	**Dyslipidemia**	**Hyper-cholesterinemia**	**Underlying disease or prior events**	**Family history**	**Medication**
**11**	Art. hypertension, palpitation and syncope		x			Hyper-tension	Father died at the age of 48 of a MI	Lisitril; Amlodipin 5mg
**12**	Tachycardia and dyspnoea							Viagra 1/week
**13**	Syncope	none				Coronary cardiopathy Stent		Aspirin cardio 100mg; Sortis 20mg
**14**	Carotic dissection	none				Stenosis A. carotis interna		Aspirin cardio 100 mg
**15**	STEMI		x	x		s/p Coronary artery bypass graft surgery; CVI		
**17**	Narrow Complex Tachykardie	none						Oestrogel Testosteron-derivative (since years)
**19**	Atrial tachycardia	none				s/p transposition of the great vessels with ventricular septal defect		Aspirin cardio 100mg; Lisitril 20mg

Neurological events

Patient number	Problem	Smoking	Hypertension	Dyslipidemia	Hyper-cholesterinemia	Underlying disease or prior events	Family history	Medication
**30**	Epileptic seizure	none				pilocytic astrocytoma grade 1		Ditropan; Magnesium; after seizure new: Keppra 500mg
**31**	Epileptic seizure	none				epileptic seizures		Tripeltherapy with Keppra, Depakine and Phenobarbital
**35**	Epileptic seizure	x (80py)	x	x		epileptic seizure		Aspirin Cardio 100 mg; Sortis 40 mg Keppra 500 mg
**36**	Epileptic seizure	x (6py)				s/p brain abscess		
**42**	Epileptic seizure	none						none
**20**	Ischemic Stroke		Borderline hypertension					
**21**	Ischemic Stroke	none						Aspirin Cardio 100 mg (primary prevention)
**23**	Ischemic Stroke		x	x		recurrent TIAs and Amaurosis fugax left side; s/p CVI left A. cerebri media	Positive family history for IS	Plavix 75mg; Atacand 8mg; Sortis 10mg; Xatral uno; Amlodipin 5mg
